# Evaluation of the Impact of Mobile Health App Vitadio in Patients With Type 2 Diabetes: Randomized Controlled Trial

**DOI:** 10.2196/68648

**Published:** 2025-05-09

**Authors:** Maxi Pia Bretschneider, Agnieszka Barbara Kolasińska, Lenka Šomvárska, Jan Klásek, Jan Mareš, Peter EH Schwarz

**Affiliations:** 1 Department of Prevention and Care of Diabetes, Department of Medicine III Faculty of Medicine Carl Gustav Carus Technische Universität Dresden Dresden Germany; 2 Vitadio s.r.o. Prague Czech Republic; 3 Paul Langerhans Institute Dresden Dresden Germany; 4 German Center for Diabetes Research Munich Germany

**Keywords:** digital health, mHealth, digital therapeutics, type 2 diabetes mellitus, DiGA

## Abstract

**Background:**

Effective diabetes management requires a multimodal approach involving lifestyle changes, pharmacological treatment, and continuous patient education. Self-management demands can be overwhelming for patients, leading to lowered motivation, poor adherence, and compromised therapeutic outcomes. In this context, digital health apps are emerging as vital tools to provide personalized support and enhance diabetes management and clinical outcomes.

**Objective:**

This study evaluated the impact of the digital health application Vitadio on glycemic control in patients with type 2 diabetes mellitus (T2DM). Secondary objectives included evaluating its effects on cardiometabolic parameters (weight, BMI, waist circumference, blood pressure, and heart rate) and self-reported measures of diabetes distress and self-management.

**Methods:**

In this 6-month, 2-arm, multicenter, unblinded randomized controlled trial, patients aged 18 years or older diagnosed with T2DM were randomly assigned (1:1) to an intervention group (IG) receiving standard diabetes care reinforced by the digital health app Vitadio or to a control group (CG) provided solely with standard diabetes care. Vitadio provided a mobile-based self-management support tool featuring educational modules, motivational messages, peer support, personalized goal setting, and health monitoring. The personal consultant was available in the app to provide technical support for app-related issues. The primary outcome, assessed in the intention-to-treat population, was a change in glycated hemoglobin (HbA_1c_) levels at 6 months. Secondary outcomes included changes in cardiometabolic measures and self-reported outcomes. Data were collected in 2 study centers: diabetologist practice in Dessau-Roßlau and the University of Dresden.

**Results:**

Between November 2022 and June 2023, a total of 276 patients were screened for eligibility, with 149 randomized to in intervention group (IG; n=73) and a control group (CG; n=76). The majority of participants were male (91/149, 61%). The dropout rate at month 6 was 19% (121/149). While both groups achieved significant HbA_1c_ reduction at 6 months (IG: mean –0.8, SD 0.9%, *P*<.001; CG: mean –0.3, SD 0.7%, *P*=.001), the primary confirmatory analysis revealed statistically significant advantage of the IG (adjusted mean difference: –0.53%, SD 0.15, 95% CI –0.24 to –0.82; *P*<.001; effect size [Cohen *d*]=0.67, 95% CI 0.33-1). Significant between-group differences in favor of the IG were also observed for weight loss (*P*=.002), BMI (*P*=.001) and systolic blood pressure (*P*<.03). In addition, Vitadio users experienced greater reduction in diabetes-related distress (*P*<.03) and obtained more pronounced improvements in self-care practices in the areas of general diet (*P*<.001), specific diet (*P*<.03), and exercise (*P*<.03).

**Conclusions:**

This trial provides evidence for the superior efficacy of Vitadio in lowering the HbA_1c_ levels in T2DM patients compared to standard care. In addition, Vitadio contributed to improvements in cardiometabolic health, reduced diabetes-related distress, and enhanced self-management, highlighting its potential as an accessible digital tool for comprehensive diabetes management.

**Trial Registration:**

German Clinical Trials Registry DRKS00027405; https://drks.de/search/de/trial/DRKS00027405.

## Introduction

Over 9.1 million adults in Germany have been diagnosed with type 2 diabetes mellitus (T2DM), and around 500,000 new cases of the disease occur in the German population every year [1,2]. This trend highlights a significant socioeconomic burden on the German health care system and underscores the urgent need for innovative solutions to provide high-quality care to this growing patient population.

Adequate management of diabetes is highly complex and challenging, necessitating a multimodal therapeutic approach to effectively control the condition and prevent associated microvascular and cardiovascular complications [3-5]. It requires a synergistic combination of lifestyle modifications, pharmacological treatment, and continuous patient education. Given that diabetes is directly influenced by various lifestyle factors, lifestyle modifications, including dietary changes, weight management, increased physical activity, smoking cessation, and psychological support, are the cornerstones of nonpharmacological management of the condition [4,6-10]. However, the demanding character of daily self-management imposes a significant burden on patients with T2DM. Adopting new healthier habits, along with the need for constant blood glucose monitoring and medication adherence, often leads to increased emotional distress and feelings of being overwhelmed [6,11-13]. In addition, it requires substantial diabetes-related knowledge and health literacy, enabling patients to make well-informed decisions about their lifestyle choices [8,14].

Physicians play a key role in providing patients with necessary, reliable information about their condition. They are also the ones who can refer patients with T2DM to structured diabetes self-management education (DSME) programs aiming at empowering individuals with diabetes by educating them on how to manage their condition effectively. While DSME has consistently been demonstrated to improve patients’ clinical [15] and psychosocial [16] outcomes, many DSME participants report that the program did not fully meet their information and support needs [14].

Similarly, the disease management program for type 2 diabetes patients, launched in Germany in 2002, despite its positive impact on quality of care [17-19], does not guarantee patients’ active involvement in the disease management in-between medical check-ups. While disease management program successfully improves the coordination of care among different health care professionals (HCPs) and promotes careful monitoring and evaluation of patient health outcomes through regular follow-up visits, individuals with diabetes may still struggle to adhere to recommended behavioral changes on a daily basis.

The lack of adherence to behavioral changes represents an important challenge in diabetes management, identified as the most significant modifiable factor compromising therapeutic outcomes [20,21]. Therefore, medical advancements that enhance patient adherence and sustain long-term motivation for recommended behavioral changes are vital for effective diabetes control. Digital solutions are being increasingly recognized for their crucial role in this context. By providing personalized, real-time support and educational resources, along with tailored feedback and easily accessible reports on glycemic control, mobile health solutions can support patients’ adherence to treatment, improve their self-management, and as a consequence, contribute to better health outcomes [20,22].

In Germany, since the introduction of the Digital Healthcare Act (Digitale-Versorgung-Gesetz) in December 2019, digital health apps (DiGAs) with clinically demonstrated health benefits can be prescribed to patients by their health care providers [23]. Vitadio is a DiGA dedicated to patients with type 2 diabetes intended to foster positive lifestyle changes and to support patients in effective self-management. This study aimed to provide evidence of the positive health care effects of Vitadio in individuals with T2DM. A 6-month, 2-arm, multicenter, randomized-controlled trial was conducted to assess whether using Vitadio is associated with a greater reduction of glycated hemoglobin (HbA_1c_) in patients with T2DM as compared to standard care. In addition, changes in patient-reported outcomes (PROs) and cardiometabolic parameters have been examined and compared between the 2 groups.

## Methods

### Study Design

**Figure 1:**
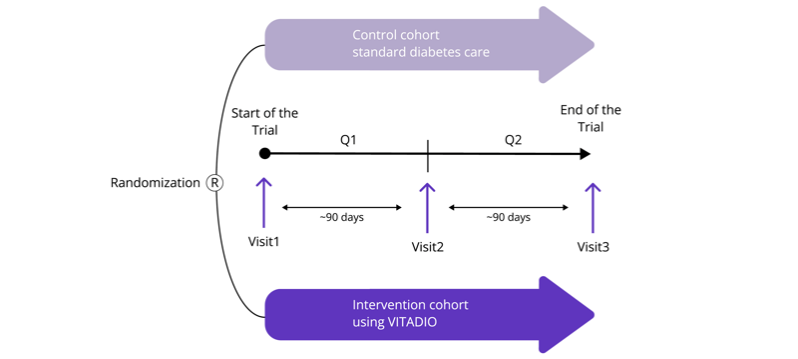
Graphical representation of the study design.

A 6-month, 2-arm, multicenter, randomized controlled trial was conducted to evaluate the effects of Vitadio on glycaemic control in patients with T2DM. Participants meeting inclusion criteria were randomly allocated in a 1:1 ratio to either the intervention group (IG) using Vitadio as an add-on to standard diabetes care or a control group (CG) receiving standard diabetes care in an ambulatory setting (refer to Figure 1; the CONSORT (Consolidated Standards of Reporting Trials) checklist is provided in Multimedia Appendix 1). The study was registered in the German Clinical Trials Registry (DRKS00027405) and was approved by the ethics committee at the Technical University of Dresden (Medical Device Regulation [MDR ff-EK-322072022]) on 07 September 2022. The clinical trial was conducted in accordance with the published principles of the International Organization for Standards 14155, MDR, German Medical Device Law Implementation Act, and applicable legislation (especially the rules for the Fast Track procedure for DiGA following Paragraph139e Social Code Book Five in Germany and the good clinical practice; refer to Multimedia Appendix 2).

### Participants

The study included participants aged ≥18 years diagnosed with T2DM. Other inclusion criteria included: (1) HbA_1c_ baseline level between ≥7.5% and ≤11%; (2) ownership of a smartphone compatible with Vitadio with internet access; (3) not having used Vitadio in the past 12 months; (4) willingness and ability to comply with all scheduled visits, laboratory tests, and lifestyle considerations; and (5) ability to provide informed consent. Exclusion criteria were (1) concurrent usage of other apps for diabetes management, (2) participation in a weight loss program, (3) presence of comorbid psychiatric conditions or cognitive impairment, (4) history of alcohol or drug abuse within the past 3 months, (5) usage of insulin pump, (6) steroid therapy within the past 3 months with the exception of topic or inhaled use (if no more than 5 times a week), (7) blood pressure ≥200 mm Hg at screening, (8) BMI >40 kg/m^2^, (9) usage of services of home health aides for blood glucose testing and insulin adjustments, (10) parallel participation in another trial or study, and (11) current or planned pregnancy during the study, breastfeeding women. 

### Recruitment

The recruitment was carried out by participating physicians in trial sites and using advertising via various marketing channels (social networks, search engines, radio, advertorials on public transport, and local newspapers). Participants recruited via consumer marketing channels were subject to an online eligibility questionnaire and an initial prescreening phone call interview. Only after positive prescreening, the participants were given an appointment at the study center at the University of Dresden or the diabetologist's practice in Dessau-Roßlau. Physicians in the study center confirmed the patients’ eligibility. All eligible participants provided written informed consent to participate in the study.

### Randomization and Masking

Eligible participants were randomly assigned to the IG or CG. Randomization was carried out at each study center based on predefined lists provided by a center for clinical trials in Dresden (“Koordinierungszentrum für klinische Studien Dresden”), where each new participant is assigned to the next free number. The “Koordinierungszentrum für klinische Studien Dresden” used a computer-based block randomization with a block size of 4 and a 1:1 ratio intervention and control. The randomization envelopes were prepared by a person not involved in the study and handed out to the respective trial sites. All participant numbers were assigned consecutively. The correct use of the randomization procedure was reviewed by the study monitor.

### Study Procedure

Following the randomization process, participants assigned to the IG received a prescription for Vitadio from the site personnel. To maintain the blinding of the sponsor, participants followed the standard app activation process by requesting the activation code from the insurance company. HCPs at the study center provided assistance in installing the application, issued a prescription or confirmation of diagnosis, and instructed participants on how to activate the Vitadio app.

Primary and secondary outcomes were collected at the study centers at 3 time points: at baseline (Visit 1), after 90 days (Visit 2), and after 180 days (Visit 3; refer to Figure 1). Additional visits for regular treatment were permitted and the treating physician had the authority to make therapeutic changes required by the patient’s clinical condition as long as they were in line with the German diabetes care guidelines. Any changes in treatment or health status of the trial participants were recorded by the study physicians during the prespecified visits (Visits 1-3) at the study centers.

### Intervention

**Figure 2:**
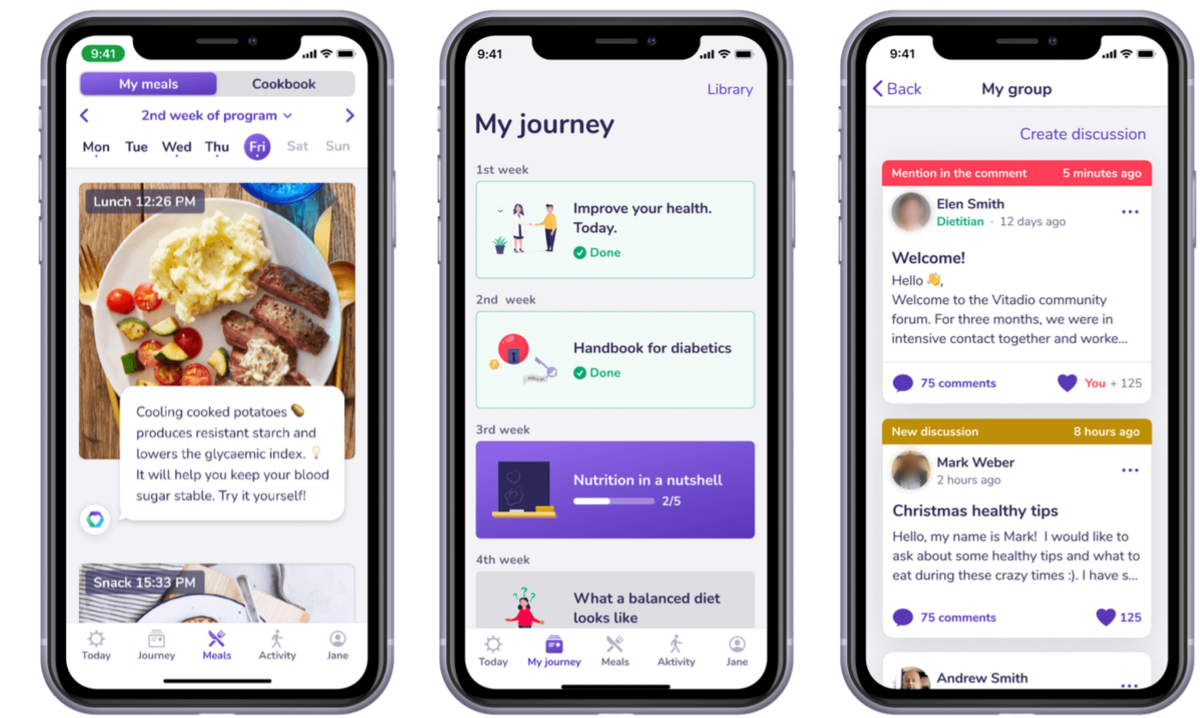
Screenshots of the Vitadio app: (a) photo-based nutrition diary with automatic feedback; (b) weekly diabetes education content; (c) peer support group for users.

Vitadio is a certified Class I medical device (low risk), in the form of a mobile app, that supports patients with diabetes in making healthy lifestyle choices and improving their self-management. Built on a multimodal therapeutic approach, Vitadio complements physician-directed therapy and helps patients in achieving their treatment goals [24,25]. The digital care program is divided into a 3-month intensive phase and the following sustain phase. It incorporates a combination of educational, motivational, and monitoring features (Figure 2). Educational materials introduce patients to the fundamentals of diabetes self-management, covering essential areas such as weight management, balanced nutrition, glycemic control, physical activity, sleep, and stress management. Daily tasks, supported by automated messages, provide patients with a structured pathway through the program and encourage consistent engagement. Personalized SMART (Specific, Measurable, Achievable, Relevant, and Time-Bound) weekly goals targeting dietary habits, activity levels, and self-care practices help reinforce positive behaviors and support the long-term sustainability of these changes. A peer support group feature fosters a sense of community, enhancing motivation and adherence to the program. Finally, the app encourages regular monitoring of physiological and lifestyle parameters to facilitate progress tracking. Participants in the IG, in addition to receiving standard diabetes care, used Vitadio for at least 180 days as part of their routine diabetes management. They had the possibility of contacting a personal consultant through the app whose primary role was to provide technical support for app-related issues.

### Comparator

The CG was instructed not to use Vitadio or any other digital health application for diabetes management during the 6-month study period. Their diabetes treatment was provided by their treating physician according to national guidelines for type 2 diabetes therapy [24] to ensure participant safety and demonstrate the additional benefits of Vitadio compared to standard care. Standard care was defined as routine care in an ambulatory setting, which includes medication adjustments but excludes the initiation of new interventions (eg, starting a lifestyle intervention) during the study. Upon completion of the trial, CG participants were invited to use Vitadio and received training from HCPs on its usage. This posttrial access to Vitadio compensated for the lack of its use during the study and addressed ethical considerations.

### Outcome Measurement

The primary objective of this study was to evaluate the efficacy of Vitadio in addition to standard care in improving glycemic control in patients with T2DM compared to standard care alone. Given that all international guidelines classify HbA_1c_ as a standard goal in diabetes management [26], the change in HbA_1c_ levels from baseline to the end of the 6-month intervention period was chosen as the primary outcome measure. To support the primary outcome analysis, the proportions of patients reaching treatment targets of HbA_1c_ ≤7.0% and ≤6.5%, as well as those who achieved the minimal clinically important difference of 0.3%, were calculated. The treatment goals were selected based on the international consensus guidelines for the management of diabetes mellitus [4,27]. To ensure that positive outcomes in the primary endpoint were attributable to the study intervention and not to other therapeutic changes occurring during the trial, changes to the diabetes-specific concomitant medication were recorded.

To provide a comprehensive overview of the effects of Vitadio on health status and outcomes in patients with T2DM, several relevant cardiometabolic and patient-reported secondary outcomes were identified. According to the German Diabetes Association Clinical Practice Guidelines, therapy goals in patients with type 2 diabetes should go beyond HbA_1c_ control and address other cardiovascular risk factors [28]. Orientation parameters for therapeutic goals have been established for weight loss (for BMI from 27-35 kg/m^2^: >5% weight reduction; for BMI >35 kg/m^2^: >10% weight reduction), systolic blood pressure (120-140 mm Hg), and diastolic blood pressure (<80 mm Hg; not <70 mm Hg) [28,29]. Therefore, changes in body weight and blood pressure have been evaluated. Moreover, considering the cardiovascular risks associated with high resting heart rate (>75-80 bpm) [30,31] and abdominal obesity [32], proxy measures of fat mass, for example, BMI and waist circumference, along with heart rate were collected.

In order to reduce the complex pharmacotherapy and the development and progression of diabetic complications, patient self-management and adherence to a healthy lifestyle are crucial [28]. The impact of the intervention on diabetes self-management was assessed using the Summary of Diabetes Self-Care Activities (SDSCA) questionnaire. The questionnaire measures levels of self-management across different components of the diabetes regimen, such as general diet, specific diet, exercise, blood-glucose testing, foot care, and smoking. Respondents report the frequency of performing relevant activities over the past 7 days [33]. To obtain a set of scores for an individual patient, the average number of days for each subscale except smoking was computed.

To evaluate the level of diabetes-related distress, the Problem Areas in Diabetes questionnaire (PAID) was administered to the participants. PAID is a self-report containing 20 items describing negative emotions (eg, fear, anger, and frustration) commonly experienced by patients with diabetes. Each question has 5 possible answers with a value from 0 to 4, with 0 representing “no problem” and 4 “a serious problem.” The scores are added up and multiplied by 1.25, generating a total score between 0-100. Patients scoring 40 or higher may be at the level of “emotional burnout” and warrant special attention [33,34].

Bearing in mind that good user experience is central to the success of interactive products, patients’ perceptions of Vitadio were assessed using the User Experience Questionnaire (UEQ). The UEQ contains 26 items rated on a 7-point Likert scale. The items are classified into 6 different dimensions: attractiveness, perspicuity, efficiency, dependability, stimulation, and novelty. Results are reported on a scale from –3 (horribly bad) to +3 (extremely good), with values between –0.8 and 0.8 representing a neutral evaluation of the corresponding scale, values >0.8 representing a positive evaluation, and values <–0.8 representing a negative evaluation [35].

Finally, the app-generated data for all participants in the IG were analyzed to better understand the patterns of usage of Vitadio and the features of the application that were most widely used.

### Statistical Analysis

The sample size calculation was performed with the program G*Power [36] and assumed a difference in HbA_1c_ reduction of 0.5% and an HbA_1c_ SD of 0.8% for the CG and 1.0% for the IG. This assumption is based on the previous results of the Czech randomized controlled trial in patients with obesity [24] and a German observational study in patients with T2DM [25]. Assuming an effect size of 0.55, a significance level of 2.5% (1-sided test), and a power of 0.8, a sample size of 69 participants per group, that is, at least 138 participants in total, was required to demonstrate a significant effect. This included an expected attrition rate of 23% [37].

Outcomes were evaluated on 2 datasets, the intention to treat (ITT) and per protocol set (PPS). PPS included all participants reaching the final study endpoint and adhering to the trial protocol without any major violations. The primary confirmatory analysis was performed based on the ITT principle. The ITT dataset encompassed all randomized patients having the baseline data for the reported variable. Missing data were imputed using the “Randomized Arm missing at randommar” [38] approach using the RefBasedMI R library. With this method, the data are considered to be missing at random, assuming the distributions of missing and observed values, conditional on observed variables, are identical. Under the missing at randommar assumption, participants were expected to keep benefiting from the treatment after dropout, with HbA_1c_ levels reflecting lifestyle changes over time, typically with a 3-month delay as a result of the Vitadio intervention.

For the primary outcome analysis, a linear mixed-effects model capturing HbA_1c_ observations over all 3 study endpoints was used. The explanatory variables included the visit number, visit site, treatment dummy, and the interaction between the visit number and treatment, which captures the treatment effect of interest, that is, the change in HbA_1c_ at the follow-up visits. Additional covariates controlled for in the model were age, sex, presence of insulin treatment, and number of years since diabetes diagnosis. In the case of ITT analysis, pooled effect sizes of 50 imputation iterations were presented. Robust SE was used and *P* values ≤.05 were considered statistically significant. For the responder analysis, the clinical relevance of treatment effects was assessed by examining the proportion of patients meeting predefined HbA_1c_ levels or changes. The significance of differences between groups was assessed via the Fisher exact test and *P* value of .05. For secondary outcomes, mean differences between the groups were compared using one-sided *t* tests at a 2.5% significance level, applicable to continuous variables. For all outcomes, the effect sizes were computed using the Cohen *d* measure.

User experience with the Vitadio app was measured using UEQ, with results being aggregated into 6D and compared against guideline thresholds of –0.8 and 0.8, respectively. Usage of the app and its features has been evaluated using internal database data over the entire study length and presented as mean (SD). The analyses of user experience and app-generated usage data were performed on the PPS dataset, focusing exclusively on the experiences of actual application users. All analyses were performed by using R version 4.3.2 or higher (R Core Team).

### Ethical Consideration

This study was approved by the ethics committee at the Technical University of Dresden (MDR ff-EK-322072022) on September 7, 2022. All eligible participants provided their personal written informed consent to participate in the study. Participants’ personal data were kept confidential and processed anonymously by assigning each participant a unique study identification number for data entry, management, and analysis. For their participation in this clinical trial, the participants received a compensation of €80 (US $90.33) at the end of the trial.

## Results

### Participants Characteristics

Participants were enrolled between November 2022 and June 2023. The first participant was included in the study on November 14, 2022. The last participant completed the study on January 2, 2024.

**Figure 3:**
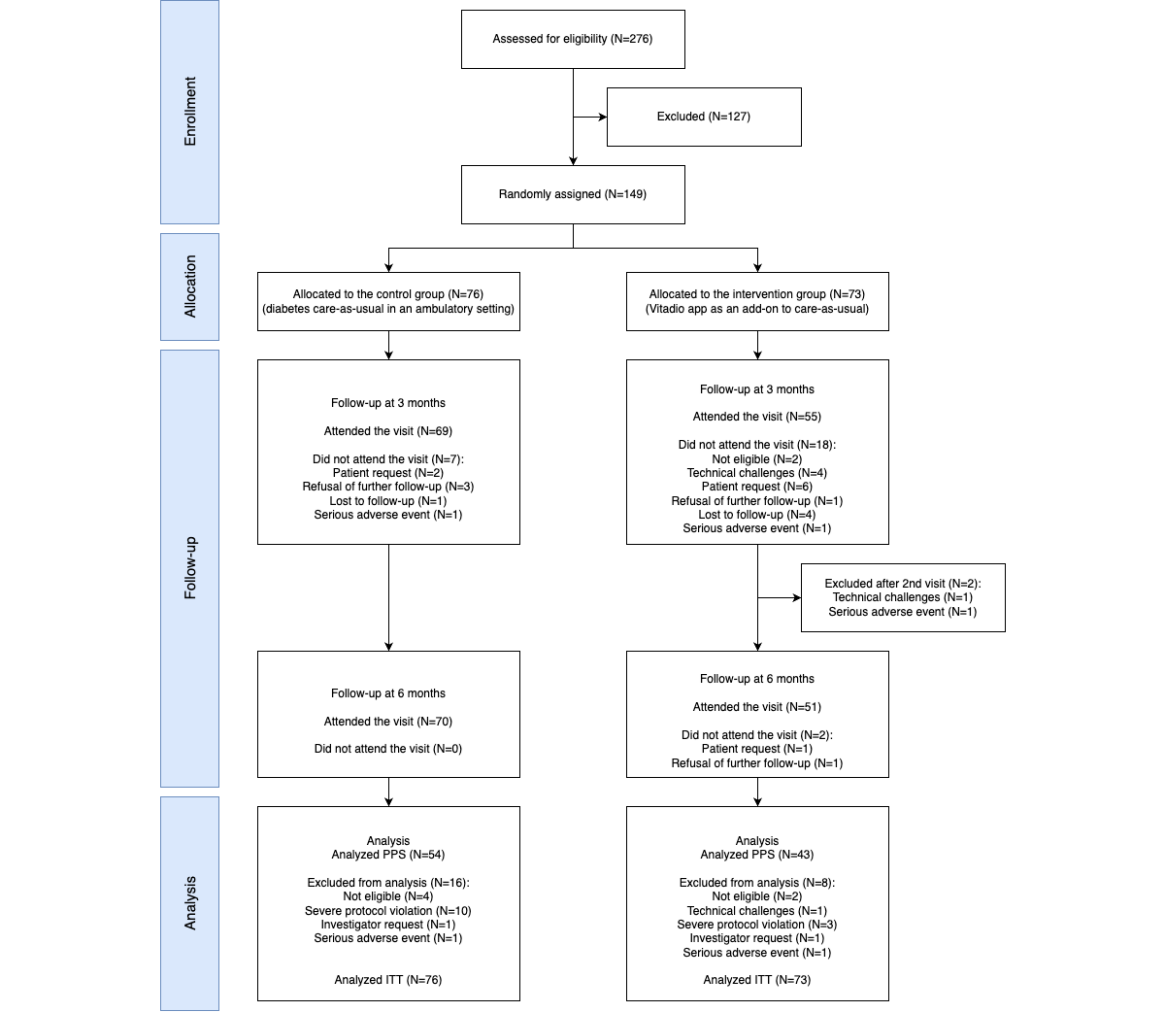
Participant flow. ITT: intention to treat; PPS: per protocol set.

A total of 276 potential participants were screened for eligibility and of those, 149 (54%) participants met the eligibility criteria and were randomized into the trial (IG: n=73, CG: n=76). The allocation was made to two study centers: Dessau-Roßlau and Dresden. In total, 124 (IG: n=69, CG: n=55) and 121 (IG: n=70, CG: n=51) participants attended the 3- and 6-month follow-ups, respectively. All in all, the PPS analysis included 97 participants. The difference in the number of patients attending the 6-month follow-up and those analyzed within the PPS is due to protocol deviations. The patient flow, including reasons for exclusions, is illustrated in Figure 3.

One participant in the CG did not attend the 3-month visit due to a serious adverse event but they did attend the final 6-month visit. This participant was withdrawn from the study due to the aforementioned adverse event.

Baseline sociodemographic and clinical characteristics are shown in Table 1. Each group comprised slightly more men (IG: 67%, CG: 57%) than women. The average age of the sample was 61 (SD 11 years) and the mean diabetes duration was 11 (SD 8 years). At baseline, there were no significant differences between the 2 groups in any of the assessed cardiometabolic parameters.

**Table 1:** Baseline sociodemographic and clinical characteristics of the sample.

Characteristics		Overall sample, (N-149)	Intervention group (N=73)	Control group (N=76)	*P* value
Male, n (%)		91 (61)	49 (67)	42 (55)	.18
Age, mean (SD)		60.6 (10.8)	61 (10.7)	60.2 (10.9)	.68
Years from diagnosis, mean (SD)		11.7 (7.5)	10.8 (7.4)	12.6 (7.6)	.14
HbA_1c_^a^ (%), mean (SD)		8.3 (0.8)	8.3 (0.7)	8.4 (0.8)	.50
Weight (kg), mean (SD)		98.3 (18.7)	98.2 (17.9)	98.3 (19.5)	.99
BMI (kg/m^2^), mean (SD)		32.2 (4.8)	32 (4.8)	32.5 (4.9)	.54
Waist (cm), mean (SD)		112.2 (12.3)	111.6 (12.5)	112.7 (12.2)	.61
Blood pressure (systolic, mm Hg), mean (SD)		151.9 (18.1)	149.8 (19.8)	154 (16.2)	.16
Blood pressure (diastolic, mm Hg), mean (SD)		89.6 (9.6)	88.9 (10.8)	90.3 (8.2)	.36
Heart rate (bpm), mean (SD)		82.8 (12.3)	81.6 (12.9)	83.9 (11.6)	.25
PAID^b^, mean (SD)		27.3 (17.6)	28.4 (18.2)	26.3 (16.9)	.48
**Summary of diabetes self-care activities questionnaire**					
	General diet, mean (SD)	4.2 (1.5)	4 (1.6)	4.3 (1.5)	.28
	Specific diet, mean (SD)	4 (1.50	3.7 (1.4)	4.3 (1.5)	.01
	Exercise, mean (SD)	3.6 (1.8)	3.7 (1.8)	3.5 (1.7)	.56
	Blood glucose, mean (SD)	3.6 (3)	3.8 (2.9)	3.5 (3)	.57
	Foot care, mean (SD)	2 (1.9)	2.3 (2)	1.7 (1.9)	.06
	Smoking, n (%)	21 (14)	10 (14)	11 (14)	1

^a^HbA_1c_: hemoglobin A_1c_.

^b^PAID: problem areas in diabetes questionnaire.

In terms of the PROs, both groups showed a similar level of diabetes-related distress during the initial testing (*P*=.48). There were also no significant differences in any but one of the aspects of diabetes self-care. The IG was characterized by lower baseline scores in the specific diet dimension of the SDSCA questionnaire (mean 3.7, SD 1.4 vs mean 4.3, SD 1.5), and the difference between the study arms was statistically significant (*P*=.01).

### Primary Outcomes

The primary objective of this study was to evaluate whether Vitadio leads to more pronounced changes in HbA_1c_ levels in patients with T2DM who followed the program for 6 months compared to a CG receiving usual diabetes care. The primary confirmatory analysis relied on a linear mixed effects model and revealed the statistically significant and clinically relevant advantage of the IG over the CG in terms of reducing the HbA_1c_ levels indicated by a model-based adjusted difference of –0.53 (SD 0.15%, 95% CI –0.24 to –0.82; *P*<.001). The effect size, as measured by Cohen *d*, was *d*=0.67 [0.33 to 1]). Treatment effects for the primary endpoint, HbA_1c_ changes at 6 months, were supported by the conducted per-protocol analysis with a difference of –0.49 (SD 0.16%, 95% CI –0.17 to –0.82; *P*=.003, *d*=0.63, 95% CI 0.22-1.05).

The mean HbA_1c_ reduction after 6 months was –0.8 (SD 0.9%; *P*<.001) in the IG and –0.3 (SD 0.7%; *P*=.001) in the CG (refer to Table S1 in Multimedia Appendix 2). The advantage of the IG could also be demonstrated by the responder analysis identifying the share of participants who achieved predefined treatment targets of HbA_1c_ ≤7.0% and ≤ 6.5%, as well as the minimal clinically important difference of –0.3% (refer to Table 2).

**Table 2:** Treatment responders – ITT population.

Responder analysis	Percentage within intervention group (n=73), %	Percentage within control group (n=76), %	*P* value (Fisher test)	Odds ratio (95% CI)
HbA_1c_ ≤ 7%	26	8	.004	4.05 (1.51-10.83)
HbA_1c_ ≤ 6.5%	11	0	.003	19.6 (1.11-346.06)
Δ HbA_1c_ ≤ –0.3%	77	52	.002	3.04 (1.5-6.17)

In IG, a significantly higher proportion of participants achieved a clinically significant reduction in HbA_1c_ of 0.3% (77%, 56/73 compared to 52%, 41/76 in CG; *P*<.05). After 6 months, 26% (19/73) of the participants in IG had reduced their HbA_1c_ level below 7%, compared to 8% (6/76) in CG (*P*=.004). The reduction below 6.5% after 6 months was achieved by 11% (8/73) of the participants in IG and none of the CG participants, representing a statistically significant difference between the groups (*P*=.003).

Relevant changes to diabetes-specific concomitant medication that occurred during the study are presented in Table 3.

**Table 3:** Changes in diabetes-specific concomitant medication during the study–PPS population^a^.

		Not present at any time	Started during the study	Increased	Decreased	Stable	Substance replaced
**OAD^b^ treatment, n (%)**							
	IG	3 (7)	0 (0)	4 (9.3)	2 (4.7)	29 (67.4)	5 (11.6)
	CG	3 (5.6)	0 (0)	12 (22.2)	3 (5.6)	32 (59.3)	4 (7.4)
	Total	6 (6.2)	0 (0)	16 (16.5)	5 (5.2)	61 (62.3)	9 (9.3)
**Insulin treatment, n (%)**							
	IG	27 (62.8)	0 (0)	5 (11.6)	2 (4.7)	9 (20.9)	0 (0)
	CG	28 (51.9)	3 (5.6)	2 (3.7)	3 (5.6)	17 (31.5)	1 (1.9)
	Total	55 (56.7)	3 (3.1)	7 (7.2)	5 (5.2)	26 (26.8)	1 (1)

^a^The percentages in parentheses express the ratio of patients in the respective group in the per protocol set (PPS).

^b^OAD: oral antidiabetic drug.

Overall, 93.8% (91/97) of the sample was treated with oral antidiabetic drugs (OAD) and 43.3% (42/97) with insulin at some point in the study. Most participants in the IG maintained a stable OAD dosage (29/43, 67.4%), whereas 22.2% (12/54) of patients in the CG required an increase in their medication dosage. Notably, although 11.6% (5/43) of patients in the IG needed to increase their insulin dosage, there were no patients who would initiate insulin therapy during the study. In comparison, only 3.7% (2/54) of participants in the CG experienced an increase in insulin dosage, but 5.6% (3/54) of the patients in this group-initiated insulin treatment during the study.

### Secondary Outcomes

The results of the analysis of the secondary outcomes are presented in Table 4. In terms of assessed cardiometabolic parameters, significant between-group differences in favor of the IG were observed for weight loss mean difference (MD=–1.33, 95% CI –2.25 to –0.42, *P*=.002), BMI (MD=–0.47, 95% CI [–0.77 to –0.16], *P*=.001) and systolic blood pressure (MD=–7.8, 95% CI –13.45 to –2.16, *P*=.004). While the IG achieved a significant reduction in waist circumference (111.65 cm to 109.86 cm, *P*<.001) and diastolic blood pressure (88.9 mm Hg to 84.28 mm Hg, *P*<.001; refer to Table S2 in Multimedia Appendix 2), no significant between-group differences were found.

**Table 4:** Between-group comparison of secondary and exploratory endpoints—intention to treat population.

Variable	Intervention group, mean (SD)			Control group (SD)			Difference in means (95% CI)		*P* value		Cohen *d* (95% CI)
	Baseline	6 months	Baseline		6 months						
Weight (kg)	98.25 (17.88)	96.05 (17.59)	98.36 (19.62)		97.49 (19.13)	–1.33 (–2.25 to –0.42)		.002		0.47 (0.14 to 0.8)	
Waist (cm)	111.65 (12.5)	109.86 (12.0)	112.65 (12.1)		112.03 (11.22)	–1.16 (–2.44 to 0.12)		.04		0.29 (–0.03 to 0.62)	
BMI (kg/m^2^)	31.98 (4.77)	31.26 (4.59)	32.5 (4.9)		32.25 (4.94)	–0.47 (–0.77 to –0.16)		.001		0.5 (0.17 to 0.83)	
Heart rate (bpm)	81.6 (12.92)	81 (12.29)	84.01 (11.63)		83.39 (11.59)	0.02 (–3.59 to 3.62)		.50		0 (–0.33 to 0.32)	
Blood pressure (systolic, mm Hg)	149.79 (19.83)	136.56 (12.65)	153.8 (16.18)		148.37 (16.57)	–7.8 (–13.45 to –2.16)		.004		0.45 (0.12,0.78)	
Blood pressure (diastolic, mm Hg)	88.9 (10.84)	84.28 (8.29)	90.12 (8.02)		87.97 (7.56)	–2.47 (–5.28 to 0.34)		.04		0.29 (–0.04,0.61)	
PAID^a^	28.36 (18.24)	19.03 (14.83)	26.32 (16.94)		23.21 (15.49)	–6.21 (–10.58 to –1.85)		.003		0.46 (0.13 to 0.79)	
SDSCA^b^– general diet	4.04 (1.56)	4.79 (0.85)	4.31 (1.52)		4.21 (1.36)	0.85 (0.4 to 1.3)		<.001		0.62 (0.28 to 0.95)	
SDSCA–specific diet	3.69 (1.44)	4.29 (1.23)	4.33 (1.55)		4.42 (1.17)	0.51 (0.1 to 0.92)		.007		0.41 (0.08 to 0.74)	
SDSCA– exercise	3.71 (1.8)	4.44 (1.42)	3.53 (1.74)		3.74 (1.6)	0.52 (0.03 to 1.02)		.02		0.34 (0.02 to 0.67)	
SDSCA– blood glucose	3.77 (2.92)	4.07 (2.72)	3.49 (3.02)		3.29 (2.97)	0.5 (–0.06 to 1.07)		.04		0.29 (–0.04 to 0.61)	
SDSCA – foot care	2.32 (1.97)	2.97 (2.14)	1.72 (1.89)		2.27 (2.11)	0.1 (–0.4 to 0.6)		.35		0.06 (–0.26 to 0.39)	

^a^PAID: problem areas in diabetes.

^b^SDSCA: summary of diabetes self-care activities.

IG participants experienced a greater reduction of diabetes-related distress than the control group (*P*=.003). Moreover, as demonstrated by the results of the SDSCA questionnaire, the IG improved their diabetes self-management in all but one (blood glucose) of the assessed dimensions (refer to Table S2 in Multimedia Appendix 2). In addition, significant between-group differences were observed in the dimensions of general diet (*P*<.001), specific diet (*P*=.007), and exercise (*P*=.02).

Vitadio was rated positively (>0.8) across most scales of the UEQ. The highest score was registered in the perspicuity dimension (mean 1.42, SD 1.06), reflecting the app’s ease of use and intuitive design. Users also appreciated the app’s attractiveness (mean 1.11, SD 1.35), along with its pragmatic qualities, such as dependability (mean 1.08, SD 1.11) and efficiency (mean 0.81, SD 0.91). The app received neutral scores in the hedonic quality aspects, specifically in stimulation (mean 0.67, SD 1.36) and novelty (mean 0.24, SD 0.93).

The analysis of app-generated data revealed that the average number of days in which the patients used the app at least once was 95 (SD 63). This implies that an average patient interacted with the app every other day. All the main features of the program showed active use by the participants: 81% (35/43) of them completed all core education materials; 83% (36/43) set at least 5 weekly goals throughout the program and 67% (29/43) of participants set their goals for every week of the intensive phase; 67% (29/43) of participants also measured their weight in the recommended, biweekly intervals; and 45% used the app to log their glycemia levels.

Patients also actively used a meal photo diary with 81% (35/43) logging their meals resulting in an average of 189 (SD 201) meal photos per participant. More than 88% (38/43) of participants used step tracking via synchronization with the Google Fit or Apple Health platform native apps. The average number of daily steps was 6647 (SD 2966) over the 6-month trial period.

## Discussion

### Principal Findings

In this prospective, 6-month randomized trial the impact of the digital lifestyle intervention Vitadio on improving glycemic control in patients with T2DM was investigated. The trial met its primary endpoint and provided evidence for the superior efficacy of Vitadio in lowering the HbA_1c_ levels in patients with diabetes compared to standard care alone. The intervention’s robust treatment effects were confirmed by the significant between-group differences in proportions of patients meeting the recommended treatment targets of HbA_1c_ ≤7.0% and ≤6.5%, and the minimal clinically important difference of –0.3%.

The analysis of diabetes-specific concomitant medication changes during the study suggests that the clinical benefits observed are primarily attributable to the Vitadio intervention itself, rather than to other therapeutic adjustments. The most notable difference between the groups was in the OAD treatment regimen. Over 20% (12/54) of patients in the CG required a dosage increase, compared to less than 10% (4/43) of the app users. In addition, although a slightly higher number of patients in the IG were prescribed increased insulin dosages (IG: n=5; CG: n=2), none of the Vitadio users needed to initiate insulin therapy during the study. Given the low number of patients who experienced changes in their insulin regimen, the impact of insulin adjustments on the clinical outcomes is likely negligible.

Effective diabetes care does not rely solely on blood glucose control. Current guidelines emphasize the importance of addressing cardiovascular risk factors in patients with diabetes [26]. It is estimated that a 10-mm Hg reduction in systolic blood pressure lowers the risk of developing major cardiovascular events by 20%, coronary heart disease by 17%, stroke by 27%, heart failure by 28%, and all-cause mortality by 13% [39]. In this study, participants in the IG not only achieved a significantly greater reduction in systolic blood pressure compared to the control condition, but they also exceeded the clinically relevant threshold of a 10-mm Hg reduction. The cardiometabolic benefits of Vitadio are further corroborated by significant improvements in all assessed cardiometabolic parameters except for resting heart rate.

In addition, the digital health application Vitadio was shown to foster positive health behaviors and improve the psychological well-being of patients with T2DM. Unlike those receiving standard diabetes care, the IG exhibited significant reductions in diabetes-related distress. This finding is clinically important, as high levels of diabetes distress are known to negatively affect self-care behaviors, medication adherence, and glycemic control [40-42]. In line with the evidence, the reduced distress aligned with the results of the SDSCA questionnaire, where Vitadio users reported significant improvements in their self-management practices. Positive changes were observed in the areas of general diet, specific diet, exercise, and foot care, resulting in significant between-group differences in all but the last dimension.

The study demonstrated several notable strengths. First, the broad inclusion criteria, which considered patients with varying durations of diabetes, both insulin- and noninsulin-treated, and with a clinically relevant and realistic HbA_1c_ spectrum, provided a not overselected patient population. The rigorous randomization procedure ensured that the groups were comparable at baseline, and the use of objective and reliable measurement methods for the primary outcomes, with assessments conducted by trained personnel at the study centers, reduced the risk of bias or measurement errors. Furthermore, the positive effects of the intervention were confirmed by both ITT and PPS analyses, underscoring the robustness of the results.

### Limitations

Some limitations of the study need to be addressed. Due to the nature of the intervention, blinding of the participants and study physicians was not feasible. While the potential expectation bias in collected PROs cannot be fully excluded, the primary outcomes, assessed through objective laboratory parameters, were unlikely to be influenced by the participants' or physicians’ awareness of the intervention received. In addition, the attrition rates between the 2 study arms varied. However, appropriate imputation methods were used to minimize the risk of introducing bias, thereby enhancing the reliability of the study's findings.

### Conclusion

These study results support the notion that Vitadio is a viable option for promoting positive lifestyle changes in patients with diabetes. As demonstrated by the analyzed app-generated data, most patients used the application regularly throughout the 6-month trial. The widespread use of educational resources and the weekly goals feature reflects increased attention to diabetes care and sustained motivation among patients. Furthermore, the application’s ease of use, demonstrated by high perspicuity scores, ensures that even those with limited technology experience can benefit from the digital therapy. This is particularly important given the association of type 2 diabetes with older age, which necessitates accommodating the specific needs of this population to ensure broad accessibility of new therapeutic tools.

## Appendix

Multimedia Appendix 1 CONSORT (Consolidated Standards of Reporting Trials) checklist EIVEL trial.

Multimedia Appendix 2 Additional materials.

## Abbreviations

CG: control group
CONSORT: Consolidated Standards of Reporting Trials
DiGA: digital health app
DSME: diabetes self-management education
HbA_1c_: glycated hemoglobin
HCP: health care professional
IG: intervention group
ITT: intention to treat
MD: mean difference
MDR: Medical Device Regulation
OAD: oral antidiabetic drugs
PAID: Problem Areas in Diabetes
PPS: per protocol set
PRO: patient-reported outcome
SDSCA: Summary of Diabetes Self-Care Activities
SMART: specific, measurable, achievable, relevant, and time-bound
T2DM: type 2 diabetes mellitus
UEQ: User Experience Questionnaire

## Data Availability

The datasets generated or analyzed during this study are available from the corresponding author on reasonable request.

## References

[1] Jacobs E, Rathmann W (2017). Epidemiologie des diabetes. Diabetologie und Stoffwechsel.

[2] Tönnies T, Hoyer A, Brinks R, Kuss O, Hering R, Schulz M (2023). Spatio-temporal trends in the incidence of type 2 diabetes in Germany. Dtsch Arztebl Int.

[3] Bain S, Cummings MH, McKay GA (2019). Multidisciplinary approach to management and care of patients with type 2 diabetes mellitus. EMJ Diabet.

[4] Davies MJ, D'Alessio DA, Fradkin J, Kernan WN, Mathieu C, Mingrone G, Rossing P, Tsapas A, Wexler DJ, Buse JB (2018). Management of hyperglycemia in type 2 diabetes, 2018. A consensus report by the American diabetes association (ADA) and the European association for the study of diabetes (EASD). Diabetes Care.

[5] Powers MA, Bardsley J, Cypress M, Duker P, Funnell MM, Fischl AH, Maryniuk MD, Siminerio L, Vivian E (2016). Diabetes self-management education and support in type 2 diabetes: A joint position statement of the American diabetes association, the American association of diabetes educators, and the academy of nutrition and dietetics. Clin Diabetes.

[6] Carpenter R, DiChiacchio T, Barker K (2019). Interventions for self-management of type 2 diabetes: an integrative review. Int J Nurs Sci.

[7] Cotter AP, Durant N, Agne AA, Cherrington AL (2014). Internet interventions to support lifestyle modification for diabetes management: a systematic review of the evidence. J Diabetes Complications.

[8] Lambrinou E, Hansen TB, Beulens JW (2019). Lifestyle factors, self-management and patient empowerment in diabetes care. Eur J Prev Cardiol.

[9] Raveendran AV, Chacko EC, Pappachan JM (2018). Non-pharmacological treatment options in the management of diabetes mellitus. Eur Endocrinol.

[10] Sneha S, Thalla S, Rischie I, Shahriar H (2021). Health internet technology for chronic conditions: review of diabetes management apps. JMIR Diabetes.

[11] Perrin NE, Davies MJ, Robertson N, Snoek FJ, Khunti K (2017). The prevalence of diabetes-specific emotional distress in people with Type 2 diabetes: a systematic review and meta-analysis. Diabet Med.

[12] Peyrot M, Rubin RR, Lauritzen T, Snoek FJ, Matthews DR, Skovlund SE (2005). Psychosocial problems and barriers to improved diabetes management: results of the cross-national diabetes attitudes, wishes and needs (DAWN) study. Diabet Med.

[13] Thoolen BJ, de Ridder D, Bensing J, Gorter K, Rutten G (2009). Beyond good intentions: the role of proactive coping in achieving sustained behavioural change in the context of diabetes management. Psychol Health.

[14] Heise M, Heidemann C, Baumert J, Du Y, Frese T, Avetisyan M, Weise S (2022). Structured diabetes self-management education and its association with perceived diabetes knowledge, information, and disease distress: Results of a nationwide population-based study. Prim Care Diabetes.

[15] Chrvala CA, Sherr D, Lipman RD (2016). Diabetes self-management education for adults with type 2 diabetes mellitus: A systematic review of the effect on glycemic control. Patient Educ Couns.

[16] Chatterjee S, Davies MJ, Heller S, Speight J, Snoek FJ, Khunti K (2018). Diabetes structured self-management education programmes: a narrative review and current innovations. Lancet Diabetes Endocrinol.

[17] Fuchs S, Henschke C, Blümel M, Busse R (2014). Disease management programs for type 2 diabetes in Germany: a systematic literature review evaluating effectiveness. Dtsch Arztebl Int.

[18] Mehring M, Donnachie E, Bonke FC, Werner C, Schneider A (2017). Disease management programs for patients with type 2 diabetes mellitus in Germany: a longitudinal population-based descriptive study. Diabetol Metab Syndr.

[19] Sönnichsen AC, Winkler H, Flamm M, Panisch S, Kowatsch P, Klima G, Fürthauer B, Weitgasser R (2010). The effectiveness of the Austrian disease management programme for type 2 diabetes: a cluster-randomised controlled trial. BMC Fam Pract.

[20] Hamine S, Gerth-Guyette E, Faulx D, Green BB, Ginsburg AS (2015). Impact of mHealth chronic disease management on treatment adherence and patient outcomes: a systematic review. J Med Internet Res.

[21] Lunde P, Nilsson BB, Bergland A, Kværner KJ, Bye A (2018). The effectiveness of smartphone apps for lifestyle improvement in noncommunicable diseases: systematic review and meta-analyses. J Med Internet Res.

[22] Hermanns N, Ehrmann D, Finke-Groene K, Kulzer B (2020). Trends in diabetes self-management education: where are we coming from and where are we going? A narrative review. Diabet Med.

[23] (2020). The fast-track process for digital health applications (DiGA) according to section 139e SGB V A guide for manufacturers, service providers and user 2020. BfArM.

[24] Moravcová K, Karbanová M, Bretschneider MP, Sovová M, Ožana J, Sovová E (2022). Comparing digital therapeutic intervention with an intensive obesity management program: randomized controlled trial. Nutrients.

[25] Bretschneider MP, Klásek J, Karbanová M, Timpel P, Herrmann S, Schwarz PEH (2022). Impact of a digital lifestyle intervention on diabetes self-management: A pilot study. Nutrients.

[26] Landgraf R, Aberle J, Birkenfeld AL, Gallwitz B, Kellerer M, Klein H, Müller-Wieland D, Nauck MA, Reuter H, Siegel E (2019). Therapy of type 2 diabetes. Exp Clin Endocrinol Diabetes.

[27] American Diabetes Association (2021). 6. Glycemic Targets:. Diabetes Care.

[28] Landgraf R, Aberle J, Birkenfeld AL, Gallwitz B, Kellerer M, Klein H, Müller-Wieland D, Nauck MA, Wiesner T, Siegel E (2022). Therapy of type 2 diabetes. Exp Clin Endocrinol Diabetes.

[29] Williams B, Mancia G, Spiering W, Agabiti Rosei E, Azizi M, Burnier M, Clement DL, Coca A, de Simone G, Dominiczak A, Kahan T, Mahfoud F, Redon J, Ruilope L, Zanchetti A, Kerins M, Kjeldsen SE, Kreutz R, Laurent S, Lip GYH, McManus R, Narkiewicz K, Ruschitzka F, Schmieder RE, Shlyakhto E, Tsioufis C, Aboyans V, Desormais I, ESC Scientific Document Group (2018). 2018 ESC/ESH guidelines for the management of arterial hypertension. Eur Heart J.

[30] Böhm M, Schumacher H, Teo KK, Lonn EM, Mahfoud F, Ukena C, Mann JFE, Mancia G, Redon J, Schmieder RE, Sliwa K, Marx N, Weber MA, Williams B, Yusuf S (2020). Resting heart rate and cardiovascular outcomes in diabetic and non-diabetic individuals at high cardiovascular risk analysis from the ONTARGET/TRANSCEND trials. Eur Heart J.

[31] Kamimura D, Tamura K (2023). Resting heart rate as a possible biomarker and target to prevent future cardiovascular disease in type 2 diabetes patients (HTR-2023-0066.R2). Hypertens Res.

[32] Després JP (2012). Body fat distribution and risk of cardiovascular disease: an update. Circulation.

[33] Toobert DJ, Hampson SE, Glasgow RE (2000). The summary of diabetes self-care activities measure: results from 7 studies and a revised scale. Diabetes Care.

[34] Snoek FJ, Bremmer MA, Hermanns N (2015). Constructs of depression and distress in diabetes: time for an appraisal. Lancet Diabetes Endocrinol.

[35] Schrepp M (2023). User Experience Questionnaire Handbook, Version 11, 2023.

[36] Faul F, Erdfelder E, Lang A, Buchner A (2007). G*Power 3: a flexible statistical power analysis program for the social, behavioral, and biomedical sciences. Behav Res Methods.

[37] Meyerowitz-Katz G, Ravi S, Arnolda L, Feng X, Maberly G, Astell-Burt T (2020). Rates of attrition and dropout in app-based interventions for chronic disease: systematic review and meta-analysis. J Med Internet Res.

[38] Cro S, Morris TP, Kenward MG, Carpenter JR (2016). Reference-based sensitivity analysis via multiple imputation for longitudinal trials with protocol deviation. Stata J.

[39] Ettehad D, Emdin CA, Kiran A, Anderson SG, Callender T, Emberson J, Chalmers J, Rodgers A, Rahimi K (2016). Blood pressure lowering for prevention of cardiovascular disease and death: a systematic review and meta-analysis. Lancet.

[40] American Diabetes Association Professional Practice Committee (2022). 5. Facilitating behavior change and well-being to improve health outcomes: standards of medical care in diabetes-2022. Diabetes Care.

[41] Hoogendoorn CJ, Schechter CB, Llabre MM, Walker EA, Gonzalez JS (2021). Distress and type 2 diabetes self-care: putting the pieces together. Ann Behav Med.

[42] Owens-Gary MD, Zhang X, Jawanda S, Bullard KM, Allweiss P, Smith BD (2019). The importance of addressing depression and diabetes distress in adults with type 2 diabetes. J Gen Intern Med.

