# Supplementary material for: Evaluation of the Impact of Mobile Health App Vitadio in Patients With Type 2 Diabetes: Randomized Controlled Trial
Source: J Med Internet Res. 2025 May 9;27:e68648. doi: 10.2196/68648 (PMC12102620; doi:10.2196/68648)
Supplement: Multimedia Appendix 2 [file jmir_v27i1e68648_app2.docx]

**Table S1.** Mean 3- and 6-month changes in HbA1c levels – ITT and PPS analyses

|  |  | **Intervention group** | | | | **Control group** | | | |
| --- | --- | --- | --- | --- | --- | --- | --- | --- | --- |
| **Dataset** | **Timepoint** | **Start** | **End** | **Change** | **p-value** | **Start** | **End** | **Change** | **p-value** |
| PPS | T_0_ – T_1_ | 8.11±0.61 | 7.47±0.87 | -0.63±0.83 | < 0.001 | 8.21±0.81 | 8.03±0.7 | -0.18±0.56 | 0.023 |
|  | T_0_ – T_2_ | 8.11±0.61 | 7.41±0.82 | -0.7±0.82 | < 0.001 | 8.21±0.81 | 8.01±0.93 | -0.2±0.75 | 0.054 |
| ITT | T_0_ – T_1_ | 8.28±0.72 | 7.6±0.82 | -0.68±0.84 | < 0.001 | 8.35±0.85 | 8.14±0.75 | -0.21±0.51 | < 0.001 |
|  | T_0_ – T_2_ | 8.28±0.72 | 7.47±0.72 | -0.8±0.89 | < 0.001 | 8.35±0.85 | 8.08±0.85 | -0.27±0.68 | 0.001 |

**Table S2.** Mean 6-month changes in secondary and exploratory endpoints – ITT and PPS analyses

|  |  | **Intervention group** | | | | **Control group** | | | |
| --- | --- | --- | --- | --- | --- | --- | --- | --- | --- |
| **Variable** | **Dataset** | **Start** | **End** | **Change** | **p-value** | **Start** | **End** | **Change** | **p-value** |
| Weight (kg) | ITT | 98.25±17.88 | 96.05±17.59 | -2.2±2.68 | < 0.001 | 98.36±19.62 | 97.49±19.13 | -0.87±2.95 | 0.013 |
|  | PPS | 96.37±15.53 | 94.71±15.7 | -1.66±3 | 0.001 | 95.68±15.75 | 94.74±15.83 | -0.94±2.57 | 0.009 |
| Waist (cm) | ITT | 111.65±12.52 | 109.86±12.02 | -1.78±3.84 | < 0.001 | 112.65±12.14 | 112.03±11.22 | -0.62±4.05 | 0.188 |
|  | PPS | 109.77±11.62 | 108.53±11.65 | -1.23±4.09 | 0.054 | 111.19±9.87 | 110.72±9.61 | -0.38±4.5 | 0.544 |
| BMI (kg/m2) | ITT | 31.98±4.77 | 31.26±4.59 | -0.72±0.89 | < 0.001 | 32.5±4.9 | 32.25±4.94 | -0.26±0.98 | 0.026 |
|  | PPS | 31.22±4.27 | 30.69±4.21 | -0.53±0.95 | 0.001 | 31.73±3.86 | 31.46±4.01 | -0.28±0.86 | 0.022 |
| Heart rate (bpm) | ITT | 81.6±12.92 | 81±12.29 | -0.6±10.6 | 0.627 | 84.01±11.63 | 83.39±11.59 | -0.62±11.58 | 0.643 |
|  | PPS | 82.65±13.11 | 81.77±13.96 | -0.88±12.7 | 0.65 | 82.52±10.88 | 82.69±12.76 | 0.17±11.89 | 0.918 |
| Blood pressure  (systolic, mmHg) | ITT | 149.79±19.83 | 136.56±12.65 | -13.23±17.09 | < 0.001 | 153.8±16.18 | 148.37±16.57 | -5.43±17.65 | 0.01 |
|  | PPS | 149.88±18.87 | 137.44±14.76 | -12.44±17.98 | < 0.001 | 153.69±16.26 | 146.83±17.46 | -6.85±19.6 | 0.013 |
| Blood pressure (diastolic, mmHg) | ITT | 88.9±10.84 | 84.28±8.29 | -4.62±9.42 | < 0.001 | 90.12±8.02 | 87.97±7.56 | -2.15±7.76 | 0.019 |
|  | PPS | 89.91±10.35 | 85±9.59 | -4.91±10.63 | 0.004 | 90.33±8.4 | 87.43±7.89 | -2.91±8.9 | 0.02 |
| PAID | ITT | 28.36±18.24 | 19.03±14.83 | -9.32±13.37 | < 0.001 | 26.32±16.94 | 23.21±15.49 | -3.11±13.51 | 0.05 |
|  | PPS | 27.38±17.48 | 17.91±15.26 | -9.48±13.03 | < 0.001 | 25.92±16.94 | 21.11±14.55 | -4.81±14.84 | 0.022 |
| SDSCA – general diet | ITT | 4.04±1.56 | 4.79±0.85 | 0.75±1.37 | < 0.001 | 4.31±1.52 | 4.21±1.36 | -0.1±1.39 | 0.517 |
|  | PPS | 4±1.34 | 4.78±0.97 | 0.78±1.26 | < 0.001 | 4.17±1.52 | 4.04±1.44 | -0.13±1.5 | 0.525 |
| SDSCA – specific diet | ITT | 3.69±1.44 | 4.29±1.23 | 0.48±1.3 | 0.002 | 4.33±1.55 | 4.42±1.17 | 0.09±1.19 | 0.513 |
|  | PPS | 3.77±1.41 | 4.31±1.47 | 0.55±1.41 | 0.015 | 4.21±1.46 | 4.31 ± 1.13 | 0.1±1.31 | 0.565 |
| SDSCA – exercise | ITT | 3.71±1.8 | 4.44±1.42 | 0.73±1.3 | < 0.001 | 3.53±1.74 | 3.74±1.6 | 0.21±1.72 | 0.301 |
|  | PPS | 3.76±1.66 | 4.4±1.53 | 0.64±1.35 | 0.003 | 3.42±1.67 | 3.67±1.73 | 0.25±1.61 | 0.255 |
| SDSCA – blood glucose | ITT | 3.77±2.92 | 4.07±2.72 | 0.3±1.52 | 0.094 | 3.49±3.02 | 3.29±2.97 | -0.2±1.95 | 0.372 |
|  | PPS | 3.52±2.93 | 3.81±2.94 | 0.29±1.48 | 0.204 | 3.58±3.01 | 3.32±3.07 | -0.26±2.29 | 0.405 |
| SDSCA – foot care | ITT | 2.32±1.97 | 2.97±2.14 | 0.65±1.55 | 0.001 | 1.72±1.89 | 2.27±2.11 | 0.55±1.53 | 0.003 |
|  | PPS | 2.1±1.94 | 3.01±2.35 | 0.91±1.65 | 0.001 | 1.77±1.92 | 2.38±2.21 | 0.6±1.71 | 0.013 |
